# Rates *versus* Subscriptions

**Published:** 1892-10-29

**Authors:** 


### Passing Topics.

### RATES *VERSUS* SUBSCRIPTIONS.

The Duke of Devonshire's observations at the meeting of the Chesterfield Hospital are remarkable chiefly for his reference to a future support of hospitals by rates as something not outside the limits of practical discussion.

His Grace confessed that at first sight it did not appear why, when there is a great and increasing tendency to throw upon public resources the support of many institutions supposed to be of advantage and benefit to the whole community, no such proposal had been made in regard to hospitals. We are not quite sure that such a proposal has not been made. We rather think a suggestion of the kind has been put forward more than once, and we cannot deny that, considered apart from all sentiment, and from the point of view of the social reformer alone, the proposal is both logical and reasonable. But the explanation why this suggestion has never been adopted by those whose position entitles them to speak on behalf of the hospitals, and whose utterances would command serious attention, is supplied by the Duke himself in a later part of his speech. It is undoubtedly due to the conviction entertained by nearly all hospital workers and hospital sympathisers alike that maintenance by rates would be fatal to the best kind of efficiency—that efficiency which does not disdain to be compassionate, a really economical consideration when sick people are concerned—and is regarded as an ultimate resort, to which recourse is to be had only when every effort to obtain a sufficiency of income by voluntary methods has irremedially failed, and the State, neglectful of its duty, has referred the hospitals to the parish.

Impersonal as some people would consider an institution to be, it is often a delicately sensitive organism. Hospitals, as represented by their responsible authorities, shrink from the receipt of parish assistance just as thoroughly as the broken-down gentility, which would rather starve than beg, and would rather beg than apply to the relieving officer. The methods of poor law relief, unhappily, are not such as to recommend them to any but the hardened or the reckless, and few more distressing proposals can well be conceived than that the hospitals, whose good offices have saved so many from the parish, should be themselves compelled to become suppliants for a form of help which carries with it, or at any rate is assumed to carry with it, an indelible stain of degradation.

That the Duke of Devonshire should have followed up his observations at Chesterfield by bestowing a handsome donation of £500 upon the local hospital, is a welcome and convincing proof of his Grace's orthodoxy, and shows that it is possible for a man, without sacrificing his reputation for a clear head and the possession of common sense, to feel at one and the same time the force of the argument that hospitals existing as they do for the benefit of all classes have an undoubted and undoubtable right to share in a general assessment, and yet to shrink from an enforcement of these legal rights at the expense of that greater right—the privilege to share the well-ordered sympathies and philanthropic offerings of all truly benevolent people. Agricola.

